# Different local, innate and adaptive immune responses are induced by two commercial *Mycoplasma hyopneumoniae* bacterins and an adjuvant alone

**DOI:** 10.3389/fimmu.2022.1015525

**Published:** 2022-12-07

**Authors:** Lisa Beuckelaere, Maarten Haspeslagh, Evelien Biebaut, Filip Boyen, Freddy Haesebrouck, Roman Krejci, Evelyne Meyer, David Gleerup, Ward De Spiegelaere, Bert Devriendt, Dominiek Maes

**Affiliations:** ^1^ Department of Internal Medicine, Reproduction and Population Medicine, Faculty of Veterinary Medicine, Ghent University, Merelbeke, Belgium; ^2^ Department of Large Animal Surgery, Anaesthesia and Orthopaedics, Faculty of Veterinary Medicine, Ghent University, Merelbeke, Belgium; ^3^ Department of Pathobiology, Pharmacology and Zoological Medicine, Faculty of Veterinary Medicine, Ghent University, Merelbeke, Belgium; ^4^ CEVA Santé Animale, Libourne, France; ^5^ Deparment of Veterinary and Biosciences, Faculty of Veterinary Medicine, Ghent University, Merelbeke, Belgium; ^6^ Department of Morphology, Imaging, Orthopedics, Rehabilitation and Nutrition, Faculty of Veterinary Medicine, Ghent University, Merelbeke, Belgium; ^7^ Department of Translational Physiology, Infectiology and Public Health, Faculty of Veterinary Medicine, Ghent University, Merelbeke, Belgium

**Keywords:** *Mycoplasma hyopneumoniae*, innate immune responses, monocytes, cell-mediated immune responses, T cells, bacterins, adjuvant, pigs

## Abstract

**Introduction:**

Enzootic pneumonia still causes major economic losses to the intensive pig production. Vaccination against its primary pathogen, *Mycoplasma hyopneumoniae*, is carried out worldwide to control the disease and minimize clinical signs and performance losses. Nonetheless, the effects of both infection with, and vaccination against *Mycoplasma hyopneumoniae* on the innate and adaptive immune responses remain largely unknown. Therefore, we conducted a study in which piglets were injected once with a commercial bacterin V1 or V2, or the adjuvant of V1 (A) to investigate their effect on local, innate and adaptive immune responses.

**Methods:**

Three weeks after vaccination, piglets were challenge infected with *M. hyopneumoniae* and euthanized four weeks later to assess vaccine efficacy *via* macroscopic and microscopic evaluation of lung lesions. Blood and broncho-alveolar lavage fluid (BAL) samples were collected to measure antibody responses, cellular immunity, BAL cytokine levels and BAL *M. hyopneumoniae* DNA load as well as cytokine secretion by monocytes.

**Results:**

After vaccination, proliferation of antigen-specific CD3^+^ T cells and a higher percentage of TNF-α^+^ CD8^+^, and TNF-α^+^ and TNF-α^+^IFN-γ^+^ CD4^+^CD8^+^ T cells was seen in V1, while proliferation of or a significant increase in cytokine production by different T cell subsets could not be observed for animals from V2. Interestingly, LPS-stimulated blood monocytes from V1 and A secreted less IL-10 on D7. After challenge, higher levels of IgA, more IL-10 and less IL-1β was detected in BAL from V1, which was not observed in V2. Animals from A had significantly more IL-17A in BAL. The macroscopic lung lesion score and the *M. hyopneumoniae* DNA load at euthanasia was lower in V1, but the microscopic lung lesion score was lower in both vaccinated groups.

**Discussion:**

In conclusion, these results indicate that the two commercial bacterins induced different local and adaptive immune responses, that the adjuvant alone can reduce anti-inflammatory innate immune responses, and that both vaccines had a different efficacy to reduce *Mycoplasma*-like lung lesions and *M. hyopneumoniae* DNA load in the lung.

## Introduction

In intensive pig production, respiratory diseases result in major economic losses. Diseases of the respiratory tract are known to be caused by several micro-organisms, including swine influenza viruses, porcine reproductive and respiratory syndrome virus, *Actinobacillus pleuropneumoniae* and *Mycoplasma hyopneumoniae*. The latter bacterium, also known as the etiological agent of porcine enzootic pneumonia, is endemically present in most pig herds and plays an important role in the porcine respiratory disease complex (1).

Vaccination against *M. hyopneumoniae* is carried out worldwide in an attempt to control the disease and minimize performance losses, clinical signs, lung lesions and treatment costs (2). Unfortunately, the current vaccines only provide partial protection and do not prevent colonization and transmission (3–5). Moreover, vaccine efficacy differs between pig herds (2), and the vaccine-induced immune responses are not yet fully elucidated. Furthermore, *M. hyopneumoniae* can activate and modulate the immune system in order to persist in the host, leading to chronic infections.

A first line of defense is provided by the host’s innate immune system, which is essential for the prevention of early pathogen replication. *Mycoplasma hyopneumoniae* is capable of enhancing the activation of host plasminogen to plasmin *via* P97/102 adhesins (6). Plasmin stimulates macrophage signaling, which results in a strong inflammatory response characterized by the presence of local pro-inflammatory cytokines (7–9). Next to the prevention of early pathogen replication, innate immune responses are also important to shape the adaptive immune responses, which involves mainly dendritic cells, as they serve as antigen presenting cells for naive T-cells, and are capable of directing the type of immune response (10). Infections with *M. hyopneumoniae* are characterized by a heavy infiltration of lymphoid cells, in particular T helper cells (11). Although cell-mediated immune responses are considered important for protection against *Mycoplasma*-induced pneumonia (10–15), they can also evolve into an excessive inflammatory response, which leads to the development of lung lesions and peribronchiolar cuffing (11, 16). On the other hand, it is known that immunoregulatory mechanisms, such as anti-inflammatory cytokines, regulatory T cells or CD8^+^ T cells, can dampen exaggerated inflammatory responses (10, 11, 17). Hence, protective immune responses against *M. hyopneumoniae* should be viewed as a delicate balance between beneficial and pathological responses (10).

Parenteral vaccination can prime the immune system, which results in higher levels of local IgG and IgA after experimental challenge (18, 19). Even though serum antibodies do not seem to be correlated with protection (18), local antibodies could be part of the mechanisms behind the prevention of clinical disease (14). To date, few studies have shown that vaccination against *M. hyopneumoniae* can also affect the cell-mediated immune responses (4, 12–14, 19–21), but only in some of these studies they evaluated the activation of different T cell subsets by measuring the cytokine production of these T cell subsets. In a few other studies, the effect of different commercial vaccines against *M. hyopneumoniae* on the cell-mediated immune responses has been compared (4, 12, 13). In the latter studies, the effect on the cell-mediated immunity was determined by delayed-type hypersensitivity (DTH) tests, lymphocyte stimulation tests, serum IFN-γ levels or by calculating the number of IFN-γ secreting cells in peripheral blood. To the authors’ knowledge, the effect of different commercial vaccines on cytokine production of CD4^+^, CD8^+^, CD4^+^CD8^+^ and CD4^-^CD8^-^ T cell subsets in an attempt to elucidate the protective immune responses that need to be triggered by *M. hyopneumoniae* vaccination has not been studied yet.

Commercially available vaccines usually consist of inactivated, whole-cell preparations, in combination with an adjuvant and excipient (2). Next to their ability to activate innate immune responses, adjuvants are also important for depot formation, enhancement of antigen presentation and they can boost vaccine-induced immune responses (22). Recently, we have shown that adjuvants can direct different T cell responses upon vaccination against *M. hyopneumoniae* (21). However, the exact mechanisms behind the functioning of adjuvants are not fully known, and the effect of an adjuvant alone on the innate immune responses and cytokine production of different T cell subsets before and after challenge infection with *M. hyopneumoniae* has not been studied.

Therefore, the aims of our study were to 1) investigate the effect of two different commercial vaccines on the local immune responses and the cytokine production by different T cell subsets and 2) assess the effect of one of the adjuvants alone on local, innate and adaptive immune responses. To this end, we measured systemic and local *M. hyopneumoniae-*specific antibodies, cytokine concentrations in BAL, cytokine production and proliferation of *M. hyopneumoniae*-specific T cells, and cytokine secretion of LPS-stimulated blood monocytes.

## Materials and methods

### Animals and experimental design

The study is in compliance with the European Directive 2010/63/EU, and was approved by the Ethics Committee of the Faculty of Veterinary Medicine and the Faculty of Bioscience Engineering, Ghent University (EC 2019-50).

Fifty-three *M. hyopneumoniae*-free piglets (Naima x Piétrain) were purchased from a commercial farrow-to-finish farm (Holzem, Luxemburg) with 210 sows and own rearing of breeding gilts. The farm has been free of *M. hyopneumoniae* for many years, based on absence of clinical signs, lung lesions at slaughter and serum antibodies. Before the start of the trial, 60 tracheobronchial swabs were collected from pigs of different ages (weaned piglets and fatteners) and tested with nested PCR (23) for the presence of *M. hyopneumoniae* DNA. All samples were negative. The farm was also free of porcine reproductive and respiratory syndrome virus and *Actinobacillus pleuropneumoniae*, and piglets were vaccinated against edema disease (Vepured^®^, Laboratorios Hipra, Girona, Spain) at one week of age.

**Figure 1:**
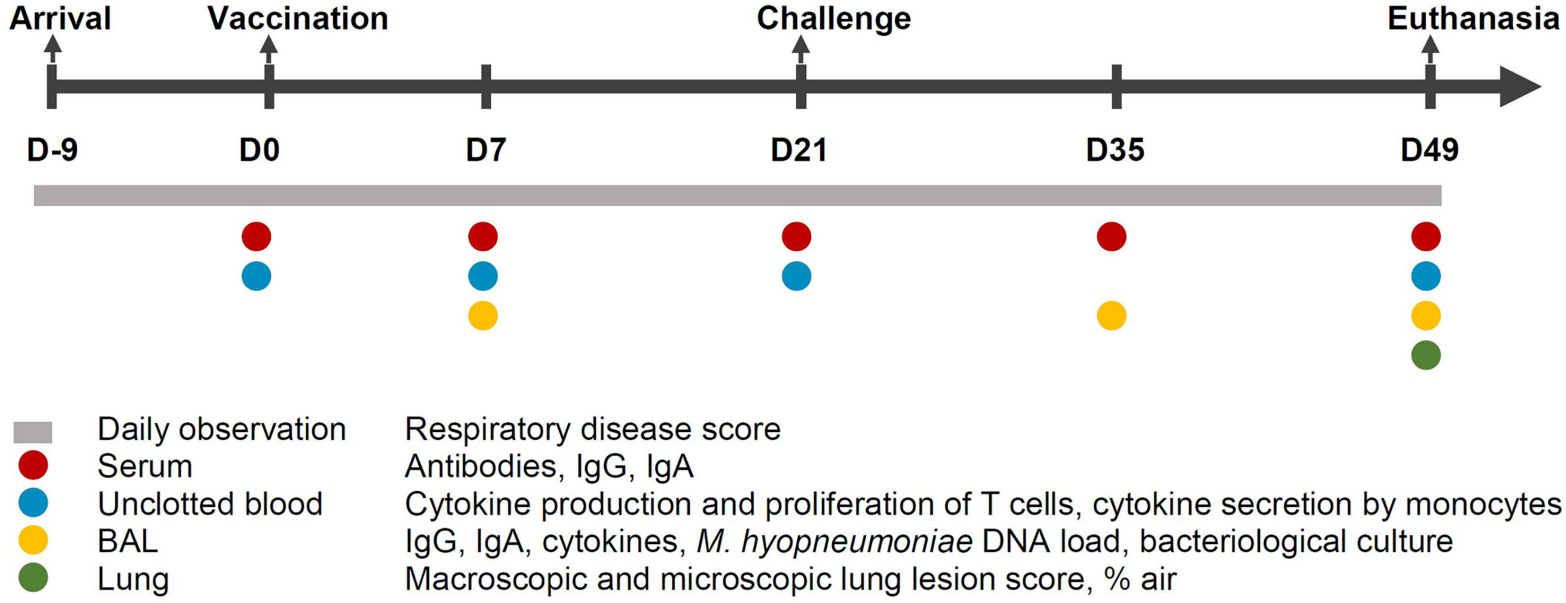
Study design. Fifty-three *M. hyopneumoniae*-free piglets were randomly allocated to one of the following groups: non-vaccinated, non-challenged sentinel group (S; n = 5); non-vaccinated, challenged control group (C; n = 12); vaccinated with commercial vaccine 1 group (V1; n = 12); vaccinated with the adjuvant of commercial vaccine 1 group (A; n = 12); vaccinated with commercial vaccine 2 group (V2; n = 12). BAL, broncho-alveolar lavage; D, days.

The experimental design and an overview of the collected samples is shown in **Figure 1**. At 28 days of age (D-9), piglets were weaned and subsequently transported to the experimental facilities. After their arrival, the weaned piglets were randomly allocated to one of the following groups: non-vaccinated, non-challenged sentinel group (S; n = 5); non-vaccinated, challenged control group (C; n = 12); vaccinated with commercial vaccine 1 group (V1; n = 12); vaccinated with the adjuvant and excipient of commercial vaccine 1 group (A; n = 12); vaccinated with commercial vaccine 2 group (V2; n = 12). Each group was housed in a separate HEPA-filtered compartment and the piglets had free access to drinking water and were fed *ad libitum* with a commercial diet without antimicrobials. During the acclimation period of 9 days, mild diarrhea was observed in all groups for which all animals were treated orally with colistin sulphate (Colivet^®^ quick-pump, Prodivet, Eynatten, Belgium) for three days, namely from D-4 till D-2 (dose 1 mL/1.3 kg body weight).

After the acclimation period, piglets were injected intramuscularly with 2 mL of vaccine 1 (V1), 2 mL of the adjuvant of vaccine 1 alone (A), 1 mL of vaccine 2 (V2) or 2 mL of physiological saline solution (S, C) (D0). Vaccine 1 is a whole-cell bacterin that consists of inactivated *M. hyopneumoniae* field isolate BA 2940-99 (antigen) and paraffin oil with *Escherichia coli* J5 non-toxic LPS (adjuvant) with thiomersal (excipient). The other bacterin consists of an *M. hyopneumoniae* J strain isolate, adjuvanted with carbomer. The adjuvant-only group received only the adjuvant with excipient of vaccine 1 (paraffin oil with *Escherichia coli* J5 non-toxic LPS and thiomersal).

On D21 and D22, all piglets were anesthetized *via* an intramuscular injection of 0.22 mL/kg body weight of a mixture of zolazepam and tiletamine (Zoletil 100^®^, Virbac, Louvain la Neuve, Belgium), and xylazine (Xyl-M^®^ 2%, VMD, Arendonk, Belgium) and subsequently inoculated endotracheally. The piglets of S received sterile modified Friis medium on both challenge days, while the piglets of the other groups were inoculated with a high virulence *M. hyopneumoniae* strain F7.2C (7 mL modified Friis medium containing 1.58 x 10^8^ CCU/mL) on D21 and a low virulence *M. hyopneumoniae* strain F1.12A (7 mL modified Friis medium containing 5.0 x 10^8^ CCU/mL) on D22 (24). Two genetically different field strains were used, as this might improve extrapolation of the obtained results to the field situation, since most pigs are simultaneously infected with two or more genetically different *M. hyopneumoniae* strains under field conditions (25). Pigs were euthanized four weeks after challenge (D49) by exsanguination after deep anesthesia with 0.30 mL/kg body weight of a mixture of zolazepam, tiletamine and xylazine.

### Sample collection

Serum samples were collected immediately before vaccination (D0), 7 days after vaccination (D7), immediately before challenge (D21), 2 weeks post-challenge (D35) and at euthanasia (D49). Serum was stored at -20°C until further analyses. Also, non-clotted blood was collected in EDTA tubes at D0, D7, D21 and D49. These samples were used immediately to determine the effect of vaccination and challenge on the cell-mediated and innate immune responses.

BAL samples were taken on D7 and D35. A catheter (Portex^®^ Dog Catheter with Female Luer Mount, Smiths Medical International Ltd., Kent, United Kingdom) was inserted in the trachea, and the lungs were flushed with 20 mL sterile phosphate buffered saline (PBS) (24). BAL samples were also collected after euthanasia (D49) by flushing the head bronchus of the right part of the lung with 20 mL sterile PBS (15). Immediately after sample collection on D49, standard bacteriological examination was performed. BAL samples were stored at -80°C until further analyses.

### Clinical and performance parameters

The animals were observed daily by the main investigator for at least 20 minutes from D-9 until D49 between 8 and 10 a.m. For each individual piglet, the severity of coughing was assessed using a respiratory disease score (RDS) (26), and abnormal clinical findings, such as loss of appetite, diarrhea, dyspnea, depression and lameness, were recorded. The RDS ranged from 0 to 6: 0 = no coughing, 1 = mild coughing after an encouraged move, 2 = mild coughing in rest, 3 = moderate coughing after an encouraged move, 4 = moderate coughing in rest, 5 = severe coughing after an encouraged move, 6 = severe coughing in rest.

### Macroscopic and microscopic lung lesions

First, the lungs were removed from the carcass at necropsy (D49) and scored for macroscopic *Mycoplasma*-like lung lesions according to the method of Hannan et al. (27). The score for the entire lung ranged from 0 (no *Mycoplasma*-like lesions) to 35 (entire lung affected). Next, a sample from the left apical, cardiac and diaphragmatic lung lobe, taken from the border of the lesion (if present), was collected from each lung for histopathological examination. These samples were first fixed in 10% neutral buffered formalin and embedded in paraffin before staining with hematoxylin and eosin. Subsequently, prepared microscopic slides were scanned using a Nanozoomer NDP slide scanner (Hamamatsu Photonics, Hamamatsu, Japan) and its viewing platform (NDP.View2). The degree of peribronchiolar and perivascular lymphohistiocytic infiltration and nodule formation (cuffing) was assessed for 10 microscopic fields (multiplication 10x) of each sample using a previously described scoring system (28). The score ranged from 1 to 5, with 1 = limited infiltration of macrophages and lymphocytes around bronchioles, with airways and alveolar spaces free of cellular exudates, 2 = light to moderate infiltrates with mild diffuse cellular exudates into airways, 3–4–5 = (mild, moderate and severe, respectively) lesions characteristic of broncho-interstitial pneumonia, centered around bronchioles but extending to the interstitium, with lymphofollicular infiltration and mixed inflammatory cell exudates. Only scores 3, 4 and 5 are suggestive for an infection with *M. hyopneumoniae*. The median microscopic lung lesion score was determined for each animal. The percentage of lung area occupied by air (% air) was also determined by analyzing 10 microscopic fields per sample with ImageJ (Bethesda Softworks, Rockville, MD, US) (24).

### Routine bacteriological culture

Ten microliters of the BAL samples collected on D49 was inoculated on a Columbia blood agar with 5% sheep blood (Oxoid, Hampshire, United Kingdom) with a *Staphylococcus pseudintermedius* streak (29). The plates were incubated for 48h at 35°C in a 5% CO_2_-enriched atmosphere to detect the presence of other respiratory bacteria. After 24 and 48h of incubation, all phenotypically different colonies were picked up and identified at the species level (score value > 2.000) by matrix-assisted laser desorption/ionization time-of-flight mass spectrometry (MALDI-TOF MS) (MALDI Biotyper, Bruker Daltonics, Bremen, Germany).

### *M. hyopneumoniae*-specific antibody responses in serum and BAL samples

A commercial blocking ELISA (IDEIA™ *Mycoplasma hyopneumoniae* EIA kit, Oxoid) was used according to the manufacturer’s instructions to measure the concentration of *M. hyopneumoniae*-specific antibodies in serum.

Both serum and BAL samples were analyzed with an indirect in-house ELISA for the presence of *M. hyopneumoniae*-specific IgG and IgA using Tween 20-extracted *M. hyopneumoniae* antigens (21). Serum samples were diluted 1:200 for IgG and 1:10 for IgA, while BAL samples from D7 and D35 were used undiluted, and BAL samples from D49 were diluted 1:10 for IgG and IgA. All serum and BAL samples were tested in duplicate. If the OD difference between both duplicates exceeded 0.05 and was more than 25% of the OD itself, this sample was retested. Positive BAL and serum samples had an average OD value higher than the cut-off value, which was calculated as the average OD of the BAL or serum samples of the sentinel group (S) + 3 times the standard deviation (SD), respectively (20).

### Cytokine immunoassays

The concentration of interferon γ (IFN-γ), interleukin 1β (IL-1β), interleukin 6 (IL-6) and interleukin 10 (IL-10) in BAL was measured using a multiplex immunoassay (Custom Porcine ProcartaPlex Multiplex Immunoassay, ThermoFisher Scientific, Waltham, MA, US) as described by the manufacturer. Broncho-alveolar lavage samples were diluted 1:2. Limits of detection (LOD) were provided by the manufacturer (1.25 pg/mL, 0.09 pg/mL, 0.43 pg/mL and 1.25 pg/mL for IFN-γ, IL-1β, IL-6 and IL-10, respectively), and limits of quantification (LOQ) were determined as the lowest concentration of the standard curve that fitted in the recovery range of 70 – 130%. Concentrations below the LOD were converted to the mean of 0 and the LOD, while concentrations below the LOQ but above the LOD were converted to the mean of the LOD and the LOQ. The test result was classified as an outlier if the concentration was lower than quartile 1 (Q1) – 1.5 x interquartile range (IQR) or higher than quartile 3 (Q3) + 1.5 × IQR. Outliers were determined within each group and day, and if present, these outliers were retested.

The concentration of IL-17A in BAL samples was measured using a commercially available kit (Kingfisher Biotech, Saint Paul, MN, US) according to the manufacturer’s protocol. Briefly, 0.25 µg capture antibody was used to coat flat-bottom 96-well plates, which were blocked after overnight incubation with 4% BSA in PBS. A standard curve was prepared by making 1:2 dilutions of the recombinant protein with 3 ng/mL as the highest concentration. Standards and a 1:2 dilution of the samples were added in duplicate and reagent diluent served as a negative control. Next, 0.1 µg detection antibody was added, followed by streptavidin-HRP (DivBioScience, Breda, The Netherlands). Finally, TMB substrate (Sigma-Aldrich, St. Louis, MO, US) was added before the reaction was stopped with stop solution (Sigma-Aldrich). The OD was measured at 650 nm and 450 nm (background measurement) with an ELISA plate reader (Multiskan GO, ThermoFisher Scientific).

### Digital PCR

Two hundred microliter of each BAL sample was used to extract DNA with a commercial kit (DNeasy^®^ Blood & Tissue kit, Qiagen, Venlo, The Netherlands) according to the manufacturer’s protocol. To measure the number of *M. hyopneumoniae* organisms without the need for a standard curve and to obtain a direct quantification, a digital PCR (dPCR) assay was developed using the forward (5’-GTCAAAGTCAAAGTCAGCAAAC) and reverse (5’-AGCTGTTCAAATGCTTGTCC) primers and the probe (5’-Cy5ACCAGTTTC-TAO-CACTTCATCGCCTCA-IAbRQSp) targeting the P102 gene as described by Marois et al. (30).

The dPCR was performed with the droplet-based Naica System (Stilla Technologies, Villejuif, France) and 16 reaction cavity Naica Opal chips (Stilla Technologies). The dPCR mixture had a reaction volume of 8 µL and contained 1X PerfeCTa^®^ Multiplex qPCR Toughmix^®^ (VWR International BVBA, Leuven, Belgium), 0.25 µM of probe, 0.5 µM of both primers, 0.1 µM fluorescein (Merck KGaA, Darmstadt, Germany), 0.4 µL FastDigest EcoRi (ThermoFisher Scientific) and 1.6 µL sample DNA. The volume of sample DNA input was optimized and possible inhibition of sample DNA was checked. High concentration samples were diluted using UltraPure™ Salmon Sperm DNA Solution (10 µg/mL, ThermoFisher Scientific) as a carrier. During each run, at least 4 non-template controls (NTC) containing reaction mixture and 1.6 µL of 10 µg/mL UltraPure™ Salmon Sperm DNA Solution were included. The Geode device (Stilla Technologies) was used for thermal cycling. Initial denaturation for 10 min at 95°C was followed by 40 amplification cycles of denaturation for 15 sec at 95°C and combined annealing/elongation for 30 sec at 60°C. The Naica Prism3 System (Stilla Technologies) was used for fluorescence readout and a fluorescence spillover compensation matrix was determined and applied to all samples using the CrystalMiner Software (Stilla Technologies). Samples were retested if there were less than 13,500 analyzable droplets or oil droplets present in the chamber. Small discrepancies in baseline fluorescence can have an influence on the number of positive droplets. Thus, the ddpcRquant method was used to apply a baseline correction to account for small shifts in baseline fluorescence (31). Next, a hard threshold was set at fluorescence 2,500 to allow partition classification as shown in **Supplementary Figure 1**. Subsequently, the limit of blank (LOB) and theoretical LOD were calculated using sixty-three NTCs. Our developed dPCR assay had a limit of blank of 2 positive partitions/well, and the theoretical limit of detection was 0.38 organisms/µL PCR mix.

### Cell-mediated immune response

In order to measure cytokines produced by T cells, peripheral blood mononuclear cells (PBMCs) were isolated from blood on D0, D7, D21 and D49, and subsequently stimulated and stained as described by others (19, 20). Briefly, 2.5 x 10^6^ cells were stimulated *in vitro* overnight for 20h with 3.125 x 10^7^ CCU of *M. hyopneumoniae* F7.2C bacterin in 0.5 mL AIM-V medium (Gibco™, ThermoFisher Scientific). Concanavalin A (10 µg/mL, Sigma-Aldrich) stimulation was used as a positive control and AIM-V medium as a negative control, to correct for background. After incubation, cells were stained with anti-CD4 (clone 74-12-4) and anti-CD8α (clone 11-295-33), and their corresponding secondary antibodies anti-mouse IgG2b FITC (Biolegend, San Diego, CA, US) and anti-mouse IgG2a PE-Cy7 (Abcam, Cambridge, United Kingdom) together with anti-CD3 DyLight755 (clone PPT3, in house labeling). Next, intracellular cytokine staining was performed using anti-human TNF-α AlexaFluor 647 (clone Mab11, Biolegend), anti-pig IFN-γ PerCP-Cy5.5 (clone P2G10, BD Pharmingen™, Becton Dickinson, Franklin Lakes, NJ, US) and anti-human IL-17A PE (clone SCPL1362, BD Pharmingen™) as described previously (20). The data were acquired with a CytoFLEX flow cytometer (Beckman Coulter, Bea, CA, US) and analyzed with CytExpert software (Beckman Coulter). The gating hierarchy is shown in **Supplementary Figure 2**.

Besides cytokine production, we also assessed T cell proliferation using a previously described protocol (20). In our study, 3.125 x 10^7^ CCU of *M. hyopneumoniae* F7.2C bacterin was used instead of *M. hyopneumoniae* J strain bacterin. Again, concanavalin A (10 µg/mL) was used as a positive control and complete cell culture medium (Dulbecco’s Modified Eagle Medium, 10% fetal calf serum, 1% penicillin/streptomycin, 1% nonessential amino acids) as a negative control, to correct for background. Data were acquired with a CytoFLEX flow cytometer and analyzed with CytExpert software. The gating hierarchy for the T cell proliferation assay can be found in **Supplementary Figure 3**.

### Cytokine secretion by blood monocytes

To investigate whether blood monocytes from V1, A and C secreted cytokines on D0, D7 or D21, monocytes were obtained by plastic adherence of PBMCs, which were seeded in a 24-well plate at 2.5 x 10^6^ cells/well and 4 wells/sample in 1 mL complete cell culture medium (Dulbecco’s Modified Eagle Medium, 10% fetal calf serum, 1% penicillin/streptomycin). Plates were incubated for 2h at 37°C with 5% CO_2_ and 90% humidity before the supernatant was discarded. Next, LPS from *Escherichia coli* O26:B6 (1 µg/well; Sigma-Aldrich) dissolved in 1 mL of complete cell culture was added to 2 wells, while 1 mL complete cell culture medium was added to the other 2 wells. Plates were incubated for another 24h. Finally, supernatant was collected, centrifuged (400×*g* at 4°C for 10 min) and stored in aliquots at -20°C until further analysis using the multiplex immunoassay described above, for which samples were diluted 1:2.

### Statistical analyses

Statistical analyses were conducted in SPSS 26 software (IBM, Armonk, NY, US). The sentinel group (S) was not included in the statistical analyses. Statistical results were considered significant when *p* ≤ 0.05.

For macroscopic lung lesion score, percentage of lung area occupied by air, serum antibody levels, BAL antibody levels, BAL cytokine concentration, BAL *M. hyopneumoniae* DNA load and cytokine secretion by monocytes, statistical comparisons were made between groups (V1, A, V2 and C) on each sampling day. First, normality and equality of variances were evaluated by Shapiro-Wilk and Levene tests, respectively. A normal distribution and equality of variances could be assumed for percentage of lung area occupied by air and therefore this parameter was analyzed using ANOVA with a Tukey’s *post hoc* test for pairwise comparisons. For all other abovementioned parameters, a Kruskal-Wallis H-test with Dunn-Bonferroni *post hoc* test was employed because parametric assumptions were violated. For these statistical analyses, the abovementioned parameters were included as dependent variable, and group as independent variable.

For T cell cytokine production and proliferation, statistical comparisons were made between groups (V1, A, V2 and C) on each sampling day. Since parametric assumptions were violated for these parameters, a Kruskal-Wallis H-test with Dunn-Bonferroni *post hoc* test was employed. The proliferation of or cytokine production by T cells was included as dependent variable, and group as independent variable. Next, the effect of sampling time was evaluated within each group using a generalized estimating equation procedure with an identity link function, accounting for repeated measurements on a pig level in an exchangeable correlation matrix and using D0 as the reference category. Data are considered relevant if significant differences were observed between the groups on a specific sampling day, and if there was a significant effect of sampling time for (at least) one of the groups on this sampling day.

The median microscopic lung lesion score was analyzed using an ordered logistic regression, with median microscopic lung lesion score as dependent variable and group as independent variable. An attempt was made to assess the influence of the vaccines and adjuvant on the RDS using a generalized estimating equations procedure with an ordinal logit link function and a subject effect of pig, however, quasicomplete separation in the data prevented model convergence and further analysis was not possible.

## Results

As expected, animals from the sentinel group (S) remained negative for serum antibodies and *M. hyopneumoniae* DNA in BAL samples throughout the study (data not shown). Three animals, one piglet from V2 on D7 and two piglets from C on D21, died during blood sampling. Also, one piglet from V1 died on D20. At necropsy, we found a necrotizing bronchopneumonia with micro-abscesses, from which *Actinomyces hyovaginalis* and *Trueperella pyogenes* were isolated. PCR testing for *M. hyopneumoniae* was negative. These four animals were excluded from RDS analysis.

### Bacterins induce different *M. hyopneumoniae*-specific antibody responses in serum and BAL

**Figure 2:**
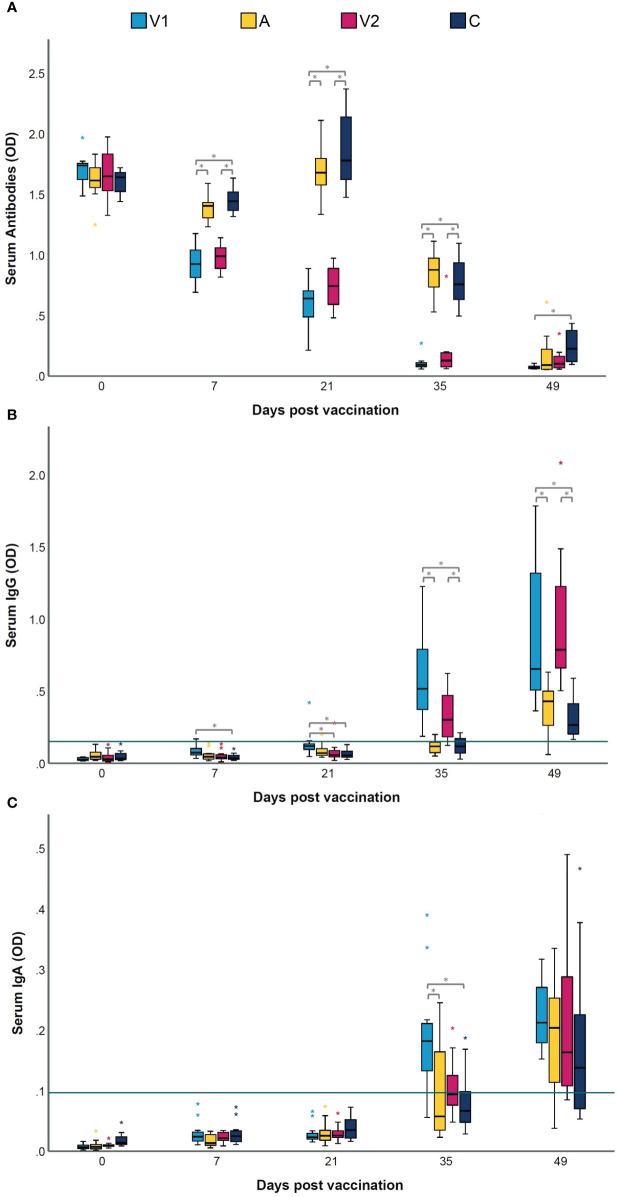
Serum antibody, IgG and IgA levels following vaccination and challenge infection. Piglets were vaccinated on D0 with V1, A, V2 or physiological saline solution (C), and animals were challenge infected on D21 and euthanized on D49. Serum antibody levels against *Mycoplasma hyopneumoniae* were measured with a commercial blocking ELISA **(A)**, and an indirect ELISA to determine IgG **(B)** and IgA **(C)** levels. In a blocking ELISA, a lower OD level corresponds with a higher antibody level. For *M. hyopneumoniae*-specific IgG and IgA, serum samples were considered as positive if they had an average OD value higher than the cut-off value, which was calculated as the average OD of the serum samples of the sentinel group (S) + 3 times the standard deviation (SD) (green horizontal line). Values are expressed as optical density (OD). For each time point, significance was calculated using a Kruskal–Wallis H-test with Dunn-Bonferroni *post hoc* test. Statistical results were considered significant when *p* ≤ 0.05 (*).

Presence of antibodies in serum and BAL was measured to confirm whether the two commercial bacterins had a different effect on antibody production. **Figure 2** shows the *M. hyopneumoniae*-specific serum antibody responses. Using a blocking ELISA, which means that a lower OD level corresponds with a higher antibody level, we observed that all piglets were negative for *M. hyopneumoniae*-specific antibodies at the start of the experiment (D0) (**Figure 2A**). Seven days after vaccination, seroconversion was observed in both vaccinated groups. Two weeks after challenge, animals from A and C also seroconverted, but significantly higher antibody levels were still measured in V1 and V2. At euthanasia, significantly more serum antibodies were found in V1 compared to C.

**Figure 3:**
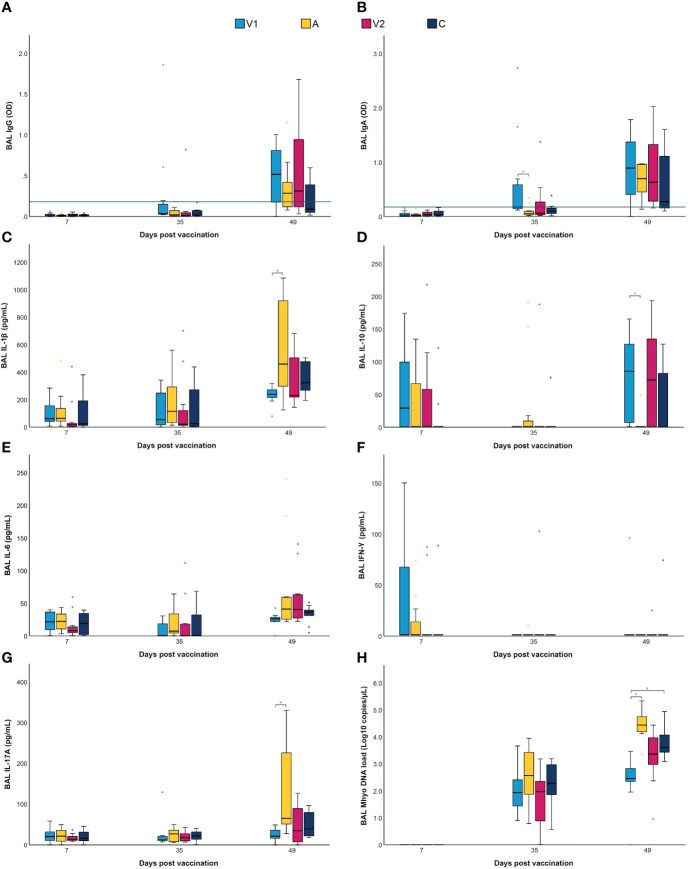
BAL IgG and IgA, cytokines and *M. hyopneumoniae* DNA load following vaccination and challenge infection. Piglets were vaccinated on D0 with V1, A, V2 or physiological saline solution (C), and animals were challenge infected on D21 and euthanized on D49. *Mycoplasma hyopneumoniae*-specific IgG **(A)** and IgA **(B)** levels were detected in BAL using an indirect ELISA, with values expressed as optical density (OD). For *M. hyopneumoniae*-specific IgG and IgA, BAL samples were considered as positive if they had an average OD value higher than the cut-off value, which was calculated as the average OD of the BAL samples of the sentinel group (S) + 3 times the standard deviation (SD) (green horizontal line). The concentration (pg/mL) of IL-1β **(C)**, IL-10 **(D)**, IL-6 **(E)** and IFN-γ **(F)** was determined using a multiplex immunoassay, and the concentration (pg/mL) of IL-17A **(G)** was determined with a commercial ELISA. The *M. hyopneumoniae* DNA load (Log_10_ copies/µL) **(H)** in BAL was investigated using a digital PCR assay targeting the P102 gene. For each time point, significance was calculated using a Kruskal–Wallis H-test with Dunn-Bonferroni *post hoc* test. Statistical results were considered significant when *p* ≤ 0.05 (*).

To obtain more insight in the antibody class induced by the *M. hyopneumoniae* vaccines, an in-house ELISA was performed on serum and BAL samples. As expected, all *M. hyopneumoniae*-specific serum IgG and IgA levels were below the cut-off value on D0 (**Figures 2B, C**). Upon vaccination, only in some animals from V1, serum IgG responses were induced, while both vaccines were unable to elicit serum IgA. However, two weeks after challenge, significantly higher serum IgA levels were detected in V1 compared to A and C. The *M. hyopneumoniae*-specific BAL IgG and IgA concentrations are shown in **Figures 3A, B**. After vaccination, there were no significant differences between the groups in BAL IgG and IgA levels. However, BAL IgA antibody levels were significantly higher in V1 compared to A two weeks after challenge, while BAL IgG levels were not affected.

In conclusion, after vaccination, seroconversion was observed in both vaccinated groups, but a serum IgG response was only detected in V1. Following challenge infection, elevated IgA levels were detected in serum and BAL from V1.

### Vaccination alters cytokine levels in lungs upon challenge infection

Next to antibodies in serum and BAL, vaccination can also lead to differences in the production of local cytokines after challenge (19, 24). Thus, we studied the effect of the two different bacterins and an adjuvant alone on cytokine levels in BAL. As shown in **Figures 3C–G**, IFN-γ and IL-6 levels in BAL did not differ between groups. Likewise, the levels of IL-1β, IL-10 and IL-17A did not significantly differ between the groups at D7 and D35. In contrast, at D49, IL-1β and IL-17A levels in BAL were significantly lower in V1 compared to A, while IL-10 levels in BAL were significantly higher in V1 as compared to A. These data imply that vaccination does not only reduce the levels of pro-inflammatory cytokines in lungs upon challenge infection, but also increases anti-inflammatory cytokine levels.

### Different T cell subsets are activated upon vaccination

**Figure 4:**
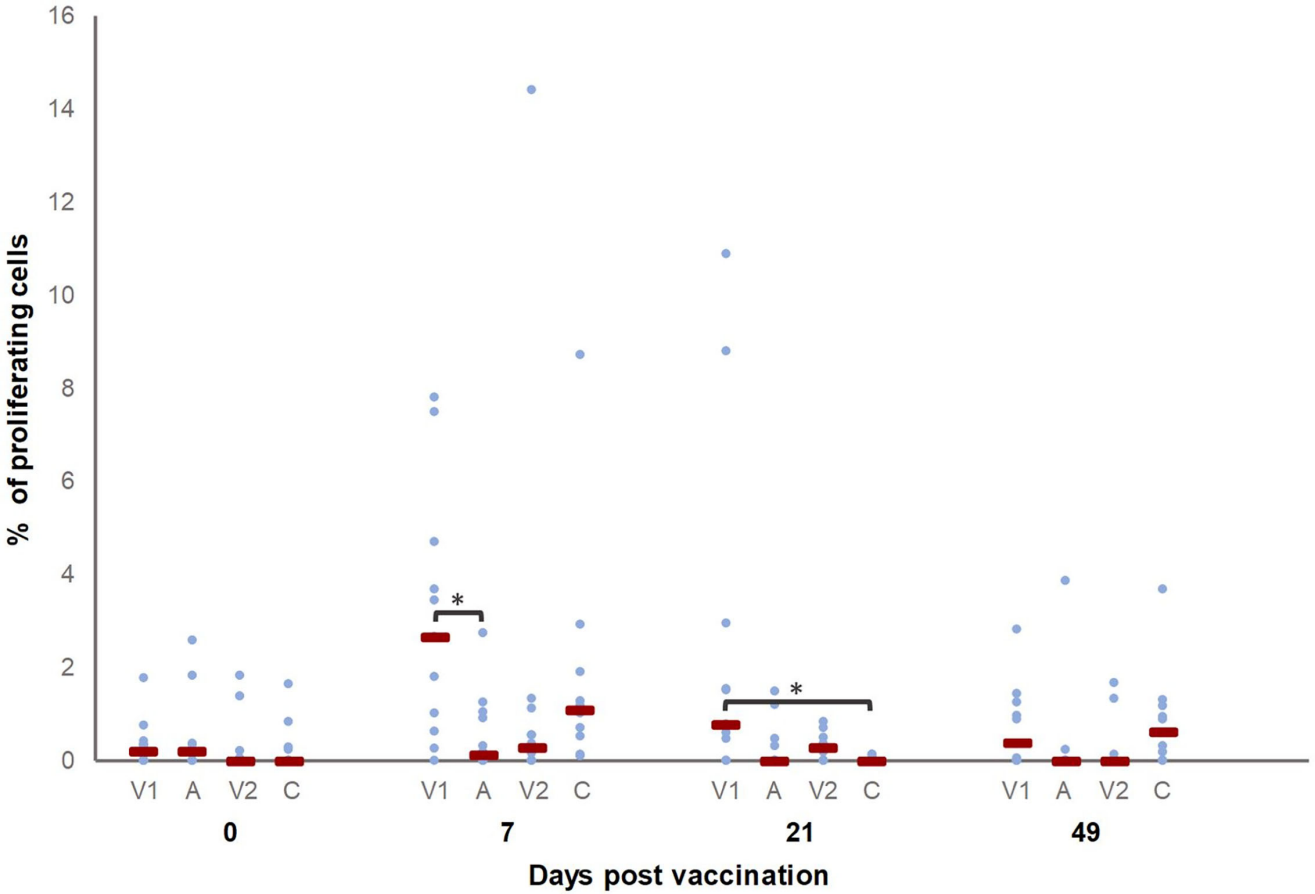
Percentage of *M. hyopneumoniae*-specific proliferating CD3^+^ T cells. Piglets were vaccinated on D0 with V1, A, V2 or physiological saline solution (C), and animals were challenge infected on D21 and euthanized on D49. The percentage of *M. hyopneumoniae*-specific proliferating CD3^+^ T cells was determined with a recall assay. First, for each time point, significant differences between groups were evaluated using a Kruskal–Wallis H-test with Dunn-Bonferroni *post hoc* test. Next, the effect of sampling time was evaluated within each group using a generalized estimating equation procedure with an identity link function, accounting for repeated measurements on a pig level in an exchangeable correlation matrix and using D0 as the reference category. Data are considered relevant if significant differences were observed between the groups on a specific sampling day, and if there was a significant effect of sampling time for (at least) one of the groups on this sampling day. Statistical results were considered significant when *p* ≤ 0.05 (*). The percentage of proliferating CD3^+^ T cells was higher on D7 and D21 as compared to D0 in V1.

To assess T cell activation, their proliferation upon recall stimulation was evaluated. T cells from all pigs proliferated upon ConA stimulation, as expected (data not shown). As shown in **Figure 4**, significantly more proliferating CD3^+^ T cells were present in V1 at D7 and D21 compared to A and C, respectively. Also, the percentage of proliferating CD3^+^ T cells was higher on D7 and D21 as compared to D0 in V1.

**Figure 5:**
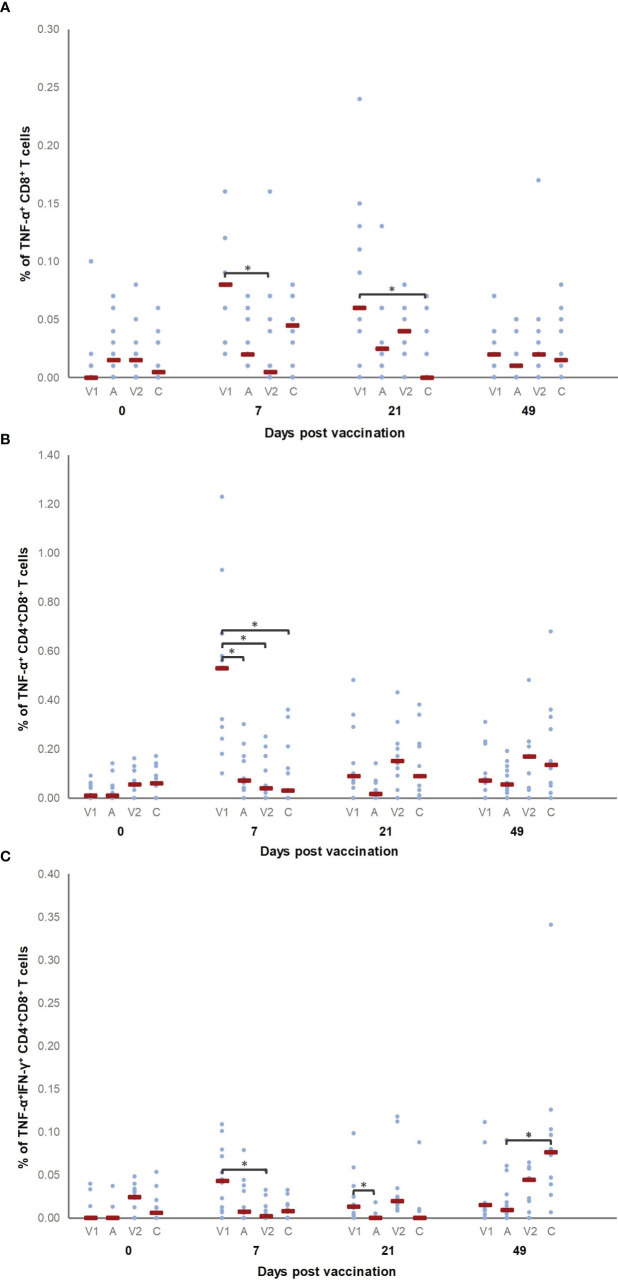
Percentage of *M. hyopneumoniae*−specific cytokine-producing T cell subsets. Piglets were vaccinated on D0 with V1, A, V2 or physiological saline solution (C), and animals were challenge infected on D21 and euthanized on D49. The percentage of *M. hyopneumoniae*−specific TNF-α^+^ CD8^+^ **(A)**, TNF-α^+^ CD4^+^CD8^+^ **(B)** and TNF-α^+^IFN-γ^+^ CD4^+^CD8^+^ **(C)** T cells was determined using a recall assay. First, for each time point, significant differences between groups were analyzed using a Kruskal–Wallis H-test with Dunn-Bonferroni *post hoc* test. Next, the effect of sampling time was evaluated within each group using a generalized estimating equation procedure with an identity link function, accounting for repeated measurements on a pig level in an exchangeable correlation matrix and using D0 as the reference category. Data are considered relevant if significant differences were observed between the groups on a specific sampling day, and if there was a significant effect of sampling time for (at least) one of the groups on this sampling day. Statistical results were considered significant when *p* ≤ 0.05 (*). A significant difference was observed for D7 and D21 as compared to D0 in V1 for the percentage of TNF-α^+^ CD8^+^ T cells, while the percentage of TNF-α^+^ CD4^+^CD8^+^ T cells was higher on D7 as compared to D0 in V1. Furthermore, the percentage of TNF-α^+^IFN-γ^+^ CD4^+^CD8^+^ T cells was higher in V1 on D7 and D21 compared to D0, and in C on D49 compared to D0.

In order to investigate whether the two vaccines activated cell-mediated immunity in a similar manner, we measured the cytokine production by different T cell subsets in a recall assay. T cells from all pigs produced cytokines upon ConA stimulation, as expected (data not shown). **Figures 5A–C** show the percentage of cytokine-producing T cell subsets for which significant differences were observed between groups before vaccination (D0), after vaccination (D7 and D21) or after challenge infection (D49). After vaccination, a higher percentage of TNF-α^+^ CD8^+^ T cells was detected in V1 as compared to V2 (D7) and C (D21), while after challenge (D49) significant differences were no longer present. For the percentage of TNF-α^+^ CD8^+^ T cells, a significant difference was observed for D7 and D21 as compared to D0 in V1.

Finally, we also evaluated cytokine production by CD4^+^CD8^+^ double positive T cells. Since in pigs these CD4^+^CD8^+^ T cells contain memory cells, an increased presence of *M hyopneumoniae*-specific CD4^+^CD8^+^ T cells upon vaccination increases the likelihood for an increased differentiation of *M hyopneumoniae*-specific memory T cells. Interestingly, V1 elicited a higher percentage of TNF-α^+^ CD4^+^CD8^+^ T cells compared to all other groups on D7. The percentage of TNF-α^+^ CD4^+^CD8^+^ T cells was also higher on D7 as compared to D0 in V1. Furthermore, the percentage of TNF-α^+^IFN-γ^+^ CD4^+^CD8^+^ T cells was higher in V1 compared to V2 and A seven days and twenty-one days after vaccination, respectively. Animals from C showed more TNF-α^+^IFN-γ^+^ CD4^+^CD8^+^ T cells compared to A four weeks after challenge. The percentage of TNF-α^+^IFN-γ^+^ CD4^+^CD8^+^ T cells was higher in V1 on D7 and D21, and in C on D49 as compared to the percentage of these T cells detected on D0 for V1 and C, respectively.

Overall, V1 induced cell-mediated immunity as evidenced by proliferating T cells, a higher percentage of TNF-α producing CD8^+^ and TNF-α and TNF-αIFN-γ producing CD4^+^CD8^+^ T cells, while a significant increase in proliferation of or cytokine production by T cells was not found in V2 after vaccination.

### The adjuvant of vaccine 1 affects LPS-induced IL-10 secretion by blood monocytes

**Figure 6:**
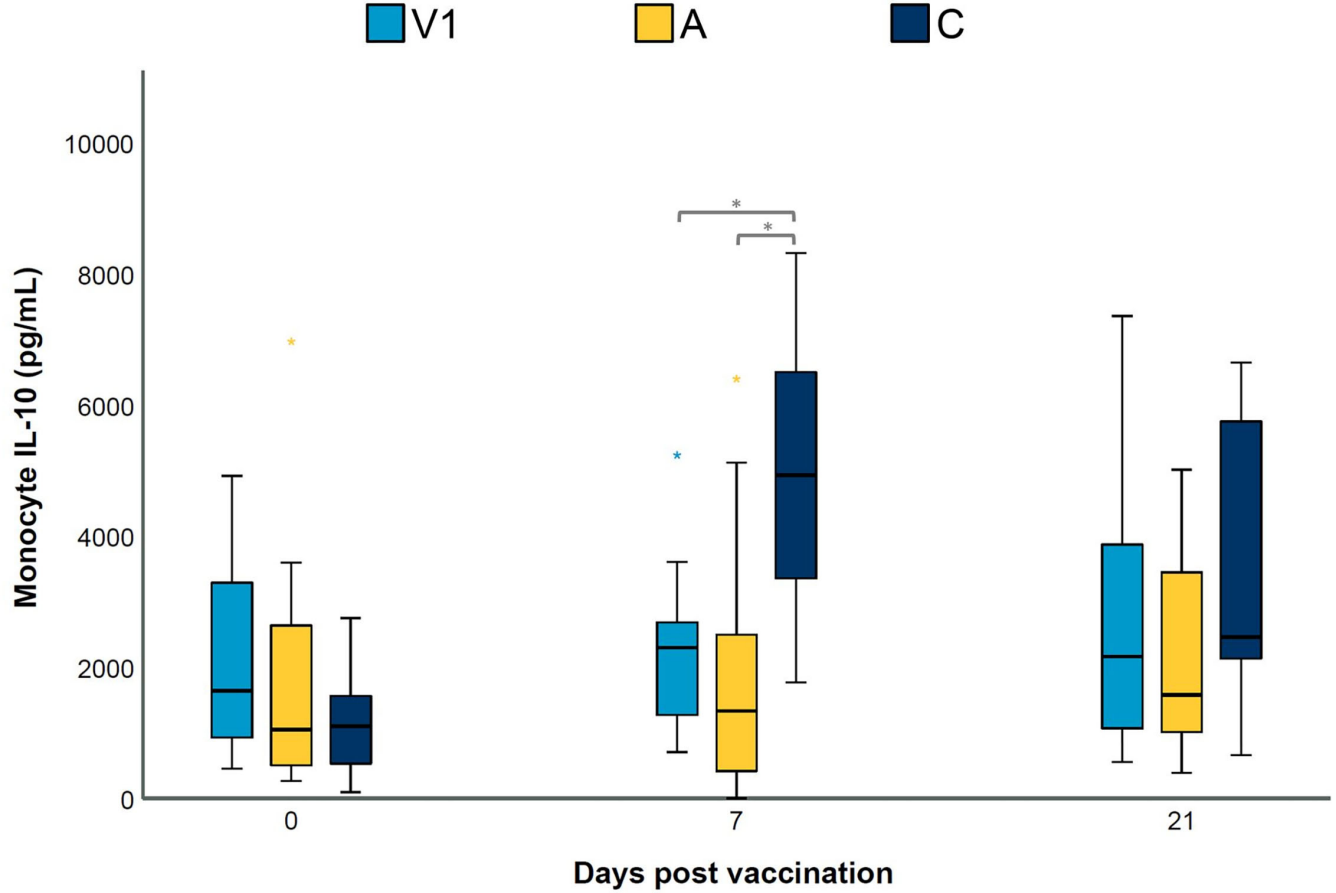
LPS-induced IL-10 secretion by blood monocytes. Piglets were vaccinated on D0 with V1, A, V2 or physiological saline solution (C), and animals were challenge infected on D21 and euthanized on D49. The concentration (pg/mL) of secreted IL-10 in cell-culture medium by LPS-stimulated blood monocytes was determined using a multiplex immunoassay. For each time point, significance was calculated using a Kruskal–Wallis H-test with Dunn-Bonferroni *post hoc* test. Statistical results were considered significant when *p* ≤ 0.05 (*).

The data presented above seem to indicate that the adjuvant alone can alter lung cytokine levels and T cell responses upon challenge infection. In an effort to elucidate the potential involved mechanisms, we investigated the effect of the adjuvant alone on cytokine secretion by blood monocytes upon LPS stimulation. As shown in **Supplementary Table 1**, monocytes from all groups on all sampling days responded to LPS stimulation by secreting IFN-γ, IL-1β and IL-6. However, no significant differences were observed between groups for these three cytokine concentrations. In contrast, IL-10 secretion of LPS-stimulated monocytes on D7 was significantly lower in V1 and A as compared to C (**Figure 6**). These data indicate that an adjuvant alone which is administered in the muscles may alter the response of circulating monocytes to LPS stimulation in pigs.

### Evaluation of vaccine efficacy parameters

To evaluate whether there are differences in efficacy between both bacterins, we studied clinical parameters, macroscopic and microscopic lung lesions, the percentage of lung area occupied by air and the *M. hyopneumoniae* DNA load in BAL.

The routine bacteriological culture of BAL collected at euthanasia (D49) revealed that no other respiratory bacteria were found in BAL from V1, V2 and C. However, *Pasteurella multocida* was detected in five out of twelve BAL samples from A. In addition, the *M. hyopneumoniae* DNA load in BAL samples was measured by dPCR. As presented in **Figure 3H**, all BAL samples collected after vaccination (D7) were negative for *M. hyopneumoniae.* Two weeks after challenge (D35), no significant differences in *M. hyopneumoniae* DNA load could be observed between groups. However, at euthanasia (D49), significantly less *M. hyopneumoniae* DNA could be detected in BAL from V1 compared to A and C.

The median macroscopic lung lesion score, median microscopic lung lesion score and average percentage of lung area occupied by air (% air) and the RDS data for each group are shown in **Supplementary Table 2**. The macroscopic lung lesion score of C observed in the current experiment is comparable to other studies (19, 24, 32). Two examples of lungs from animals with and without Mycoplasma-like lesions are shown in **Supplementary Figure 4**. There was a significant difference in the macroscopic lung lesion score between V1 and all other groups. On the other hand, the microscopic lung lesion score differed significantly between V1 and C, and V2 and C. In contrast, there was no significant difference between the groups in the percentage of lung area occupied by air. Despite differences in lesion score, a quasi-complete separation in the RDS data prevented model convergence and further statistical analysis for this parameter was not possible.

In conclusion, animals from V1 showed a lower *M. hyopneumoniae* DNA load in BAL and a significant reduction of the macroscopic lung lesions, while in both V1 and V2 we observed less microscopic lung lesions compared to C. These findings indicate that both bacterins differ in their efficacy and capacity to reduce *Mycoplasma*-like lung lesions.

## Discussion

We have shown that two commercial bacterins, which are frequently used in European countries and are based on different *M. hyopneumoniae* strains and different adjuvants, have (1) a different efficacy and capacity to reduce *Mycoplasma*-like lung lesions and (2) a different effect on local and adaptive immune responses. The different efficacy of these bacterins might enable to correlate vaccine-induced immune responses with enhanced efficacy. In our study, we also included a group of animals which received the adjuvant of V1, a vaccine for which a significant reduction of the respiratory disease score and macroscopic lung lesion score was already observed (24). Even though it would have been interesting to include more groups receiving different adjuvants, this was not feasible in the current study due to the labor intensive nature of the experiment.

In our study, less macroscopic lung lesions were seen in V1, and the load of *M. hyopneumoniae* DNA, which is indicative for shedding of the bacterium, was also lowest in this group. These results suggest that V1 induced immune responses enabling the piglets to better clear the infection. Indeed, two weeks after challenge, local *M. hyopneumoniae*-specific IgA levels were only elevated in V1, and less pro-inflammatory and more anti-inflammatory cytokines were detected in BAL from V1 at euthanasia. Furthermore, only vaccine 1 appeared to have an effect on the proliferation of and cytokine production by different *in vitro M. hyopneumoniae* stimulated T cell subsets. Interestingly, we also observed that (3) the adjuvant of vaccine 1 alone can have an influence on the anti-inflammatory response of circulating innate immune cells to LPS stimulation. We believe that more research, including on other adjuvants, is desirable to further elucidate the involved mechanisms and the importance of the adjuvant in the induction of protective immunity.

Others have shown that commercial vaccines can induce *M. hyopneumoniae*-specific serum antibodies, and that the serological response (4) and percentage of seroconverting animals (33) can differ between vaccines. These differences in serological response might be due to differences in adjuvant, antigen dose, level of antigen expression, or even individual animal responses (21, 34). After vaccination, we were able to detect serum antibodies in both bacterin groups using a commercial blocking ELISA for *M. hyopneumoniae*, but only a limited response in *M. hyopneumoniae*-specific serum IgG and no elevation of *M. hyopneumoniae*-specific serum IgA was seen. The lower sensitivity of the in-house IgG ELISA could be explained by the fact that serum samples were less diluted for the commercial ELISA compared to our in-house IgG ELISA. Nevertheless, only in V1, we detected *M. hyopneumoniae*-specific serum IgG responses after vaccination and *M. hyopneumoniae*-specific serum IgA responses two weeks after challenge. Thus, differences were detected in *M. hyopneumoniae*-specific serum antibodies between both bacterins. However, since *M. hyopneumoniae*-specific serum antibodies probably play a minor role in protective immunity (4, 18), also local mucosal immunity and cell-mediated immune responses were characterized.

Local mucosal IgA antibodies are considered important to prevent the adherence of *M. hyopneumoniae* to the ciliated epithelium of the respiratory tract (14, 35). Parenteral vaccination might prime the immune system so higher levels of local IgG and IgA are generated after challenge infection (18, 19). Indeed, in our study, both bacterins did not induce a detectable BAL IgA response after vaccination, but most animals from V1 had significantly higher BAL IgA concentrations two weeks after challenge, which indicates priming of the immune system by vaccination. These local antibodies could be part of the mechanisms behind the prevention and/or control of clinical disease (14), however, other parts of the immune system may also be important for protection.

A strong inflammatory response, which is characterized by the presence of local pro-inflammatory cytokines, is triggered after colonization of *M. hyopneumoniae* of the lower respiratory tract (7–9). These host immune responses can help clear *M. hyopneumoniae* infections, but an excessive inflammatory response can also lead to the development of lung lesions and peribronchiolar cuffing (16). Therefore, immune responses should be viewed in light of a delicate balance between beneficial responses and those contributing to disease pathogenesis (10). It has been shown by others that vaccination can have an influence on the local cytokine levels after challenge (14, 21). In our study, we detected a significant reduction of the pro-inflammatory cytokine IL-1β and an increased concentration of the anti-inflammatory cytokine IL-10 after challenge in BAL from V1. It is possible that the reduction of macroscopic lung lesions in V1 was at least partially due to the combination of a suppressed pro-inflammatory and an elevated anti-inflammatory response. Remarkably, in A, an elevated IL-17A concentration in BAL could be detected after challenge infection. Possibly, IL-17A levels in BAL in this group were higher due to the high *M. hyopneumoniae* DNA load. It should also be noted that in A, IL-10 levels in BAL were close to 0 and significantly lower than V1. One might assume that this predominant pro-inflammatory response leads to a higher degree of lung lesions, yet the median macroscopic lung lesion score was not statistically different from the control group. We observed that an intramuscular injection of this adjuvant alone can result in long-term effects on the systemic innate immune responses. However, more research is needed to further elucidate the exact mechanisms behind this observation, and to study the effect of other adjuvants on the concentration of local cytokines.

Cell-mediated immune responses, like antigen-specific Th1 and Th17 cells, mainly derived from CD4^+^CD8^+^ T cells, as well as CD8^+^ T cells are considered important for the protection against a *Mycoplasma*-induced pneumonia (10–15, 36). However, an exaggerated inflammatory response has also been linked to the development of lung lesions (11), making it difficult to pinpoint effector T cell subsets that are involved in protection against *M. hyopneumoniae* infections. Vaccination of pigs with commercial *M. hyopneumoniae* bacterins has been shown to induce the presence of several T cell subsets in blood and secondary lymphoid tissues (15, 37). Based on IFN-γ levels in serum, DTH tests and lymphocyte stimulation assays, a previous study hinted that there might be differences between commercial bacterins regarding the effect on the cell-mediated immune responses (12). We observed that V1 induced antigen-specific CD3^+^ proliferating T cells on D7 and D21, which indicates that *M. hyopneumoniae*-specific T cells are generated after vaccination.

We were not able to detect Th17 responses or cytokine production by CD4^+^ T cells after vaccination, corroborating our previous results (19, 21). Pro-inflammatory Th1 responses, which mainly involve IFN-γ or TNF-α producing CD4^+^ T helper cells, and Th17 responses are considered important for protection against infections with extracellular pathogens (11, 19, 38, 39). Recently, the long-held belief that *M. hyopneumoniae* is a strict extracellular pathogen was challenged by Raymond et al. (40), who have provided some evidence that *M. hyopneumoniae* might invade porcine respiratory epithelial cells *in vivo*. Despite the absence of Th1 and Th17 responses, we did detect less macroscopic lung lesions in V1, which indicates that other T cell subsets play an important role in the protection.

Previous research has shown that stimulation of naïve CD4^+^ T helper cells can lead to maturation to CD4^+^CD8^+^, a T cell subset that, among other cells, contains antigen-specific memory function cells (41–43). Unlike human and mouse peripheral blood, blood of adult pigs contains a substantial population of CD4^+^CD8^+^ T cells, which increases with ageing (41, 44–46). After vaccination, we detected TNF-α and TNF-αIFN-γ producing CD4^+^CD8^+^ T cells in V1, which suggests that the CD4^+^ T cells of these animals probably matured after vaccination. For animals from V2, an increase in the cytokine production by CD4^+^CD8^+^ T cells could not be observed.

Animals from V1 had a significant increase in the percentage of TNF-α^+^ CD8^+^ T cells after vaccination, while a significant increase in cytokine production of CD8^+^ T cells was not observed in V2. As mentioned before, Raymond et al. (40) showed that a subpopulation of *M. hyopneumoniae* can bind to β1 integrin, which allows *M. hyopneumoniae* to invade and reside in porcine epithelial cells. Intracellular *M. hyopneumoniae* are able to survive fusion with lysosomes and escape into the cytosol, and the bacteria were observed re-entering the extracellular milieu *via* recycling endosomes. Next to their ability to dampen exaggerated inflammatory responses (11), CD8^+^ T cells are primarily known as cytotoxic T cells, which recognize and kill host cells that are infected by intracellular pathogens. This might explain the significantly lower macroscopic lung lesion scores from V1, as animals from this group possessed CD8^+^ T cells which are able to respond upon intracellular *M. hyopneumoniae*.

The innate immune system is a first line of defense against pathogens, including *M. hyopneumoniae* and is essential for protection, since it can prevent early pathogen replication. Upon recognition of *M. hyopneumoniae* by innate immune cells, a strong inflammatory response is triggered which enables the host to control early infection and which in turn drives the induction of adaptive immune responses. The adjuvant used in vaccine 1 is a mineral oil-in-water emulsion (paraffin) with *Escherichia coli* J5 non-toxic LPS. Oil-in-water emulsions are usually well tolerated and induce strong short-term immune responses. In a previous study (21), we observed an elevated rectal temperature in the group that received vaccine 1 on the day of vaccination. Furthermore, oil-in-water adjuvants are known to affect innate immune responses and can enhance humoral and cellular responses (22, 47).

Administration of low doses of LPS, followed by homologous LPS restimulation has been shown to result in a depressed inflammatory immune response (48, 49). However, in our study, we did not detect significant differences in the secretion of pro-inflammatory cytokines by blood monocytes in V1 or A compared to C. This finding suggests tolerance of blood monocytes to LPS was not present in our study. Furthermore, it has been demonstrated that innate immune cells can be trained after infection or vaccination in an antigen independent manner (50). Indeed, we found that LPS-stimulated blood monocytes from V1 and A secreted significantly less anti-inflammatory IL-10 seven days after vaccination. Interestingly, this adjuvant alone affected circulating blood monocytes, which are present far from the injection site, and this response was detected after the adjuvant itself had probably disappeared. We hypothesize that the blood monocytes in our study were primed to respond in a less anti-inflammatory way, which might be beneficial to trigger protective immune responses. However, further research is needed to elucidate the involved mechanisms and to understand the importance of this and other adjuvants in the induction of protective immunity against *M. hyopneumoniae* and potentially other pathogens.

In conclusion, our study showed that different *M. hyopneumoniae* bacterins can have a different effect on both local and adaptive immune responses, and that the adjuvant alone can alter the response of circulating innate immune cells to LPS stimulation. V1 showed the highest efficacy to reduce *Mycoplasma*-like lung lesions and *M. hyopneumoniae* DNA load in BAL, and induced a strong TNF-α^+^ CD8^+^ and TNF-α^+^ and TNF-α^+^IFN-γ^+^ CD4^+^CD8^+^ T cell response after vaccination. Furthermore, IgA in BAL after challenge was only detected in this group. Therefore, we propose that vaccines against *M. hyopneumoniae* should induce, activate and enhance these immune responses to prevent lung lesions. Hence, our findings might support the development of next generation vaccines able to elicit protective immunity against *M. hyopneumoniae* infections.

## Data availability statement

The original contributions presented in the study are included in the article/**Supplementary material**. Further inquiries can be directed to the corresponding author.

## Ethics statement

The animal study was reviewed and approved by the Ethics Committee of the Faculty of Veterinary Medicine and the Faculty of Bioscience Engineering, Ghent University (EC 2019-50).

## Author contributions

LB and EB performed the animal experimentation, lab work and data analyses. MH performed the statistical analyses, and EM, DG and WS assisted with specific laboratory assays. FB, FH, RK, BD and DM designed and supervised the overall project. All authors contributed to the article and approved the submitted version.
