# Supplementary figures and images for: Different local, innate and adaptive immune responses are induced by two commercial Mycoplasma hyopneumoniae bacterins and an adjuvant alone
Source: Front Immunol. 2022 Dec 7;13:1015525. doi: 10.3389/fimmu.2022.1015525 (PMC9768447; doi:10.3389/fimmu.2022.1015525)

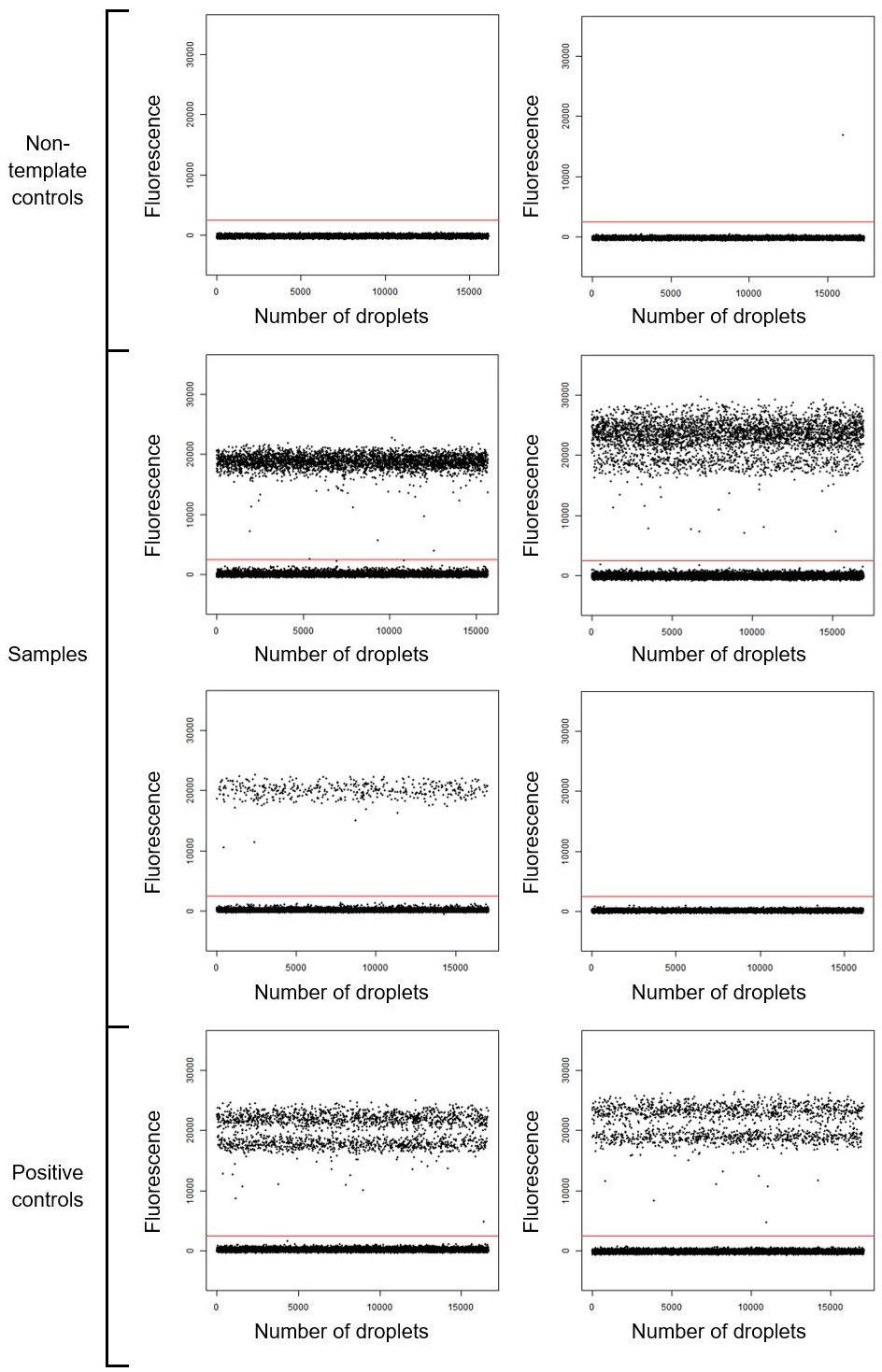

Supplement: Supplementary file 1 [file Image_1.jpeg]

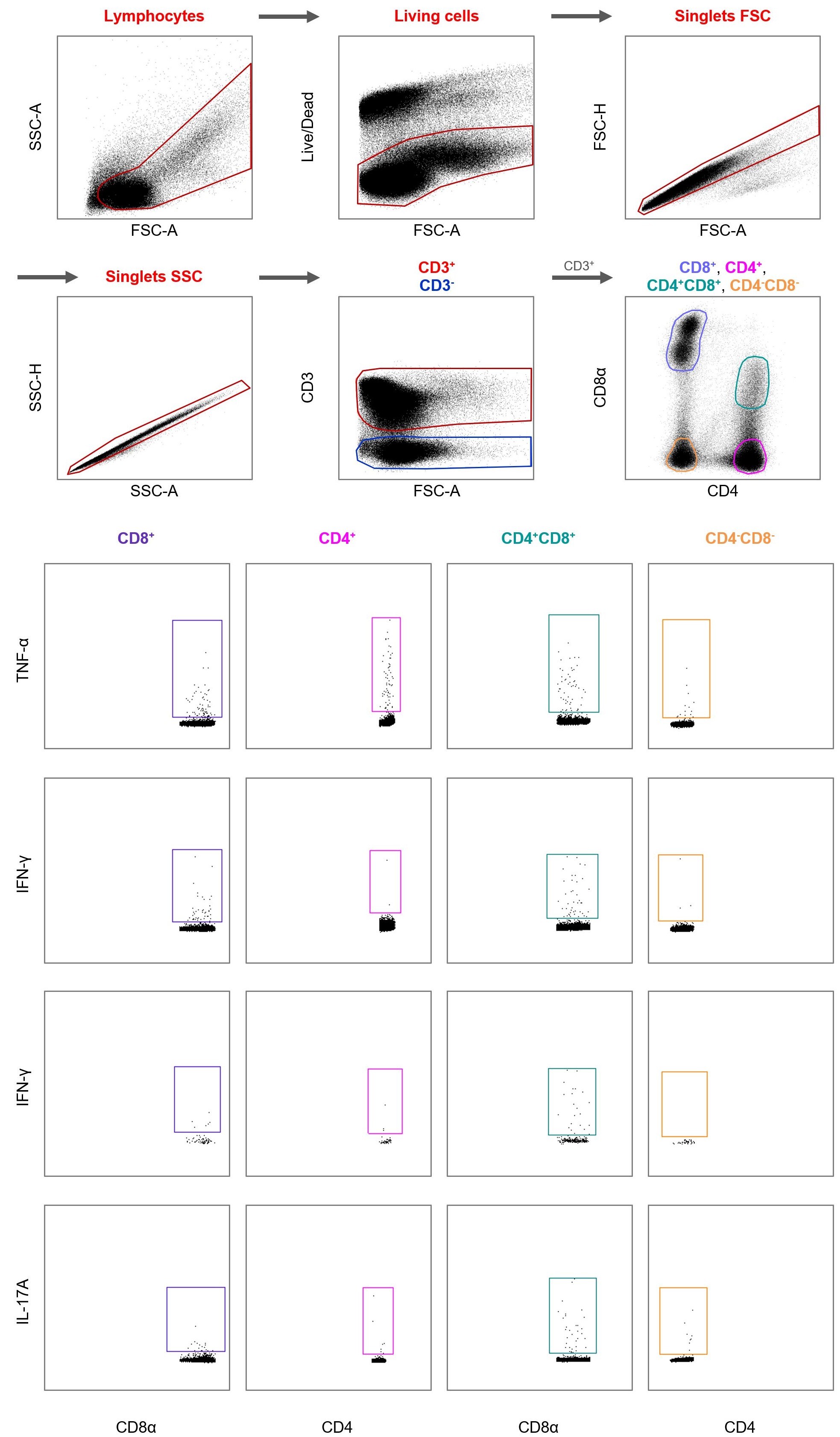

Supplement: Supplementary file 2 [file Image_2.jpeg]

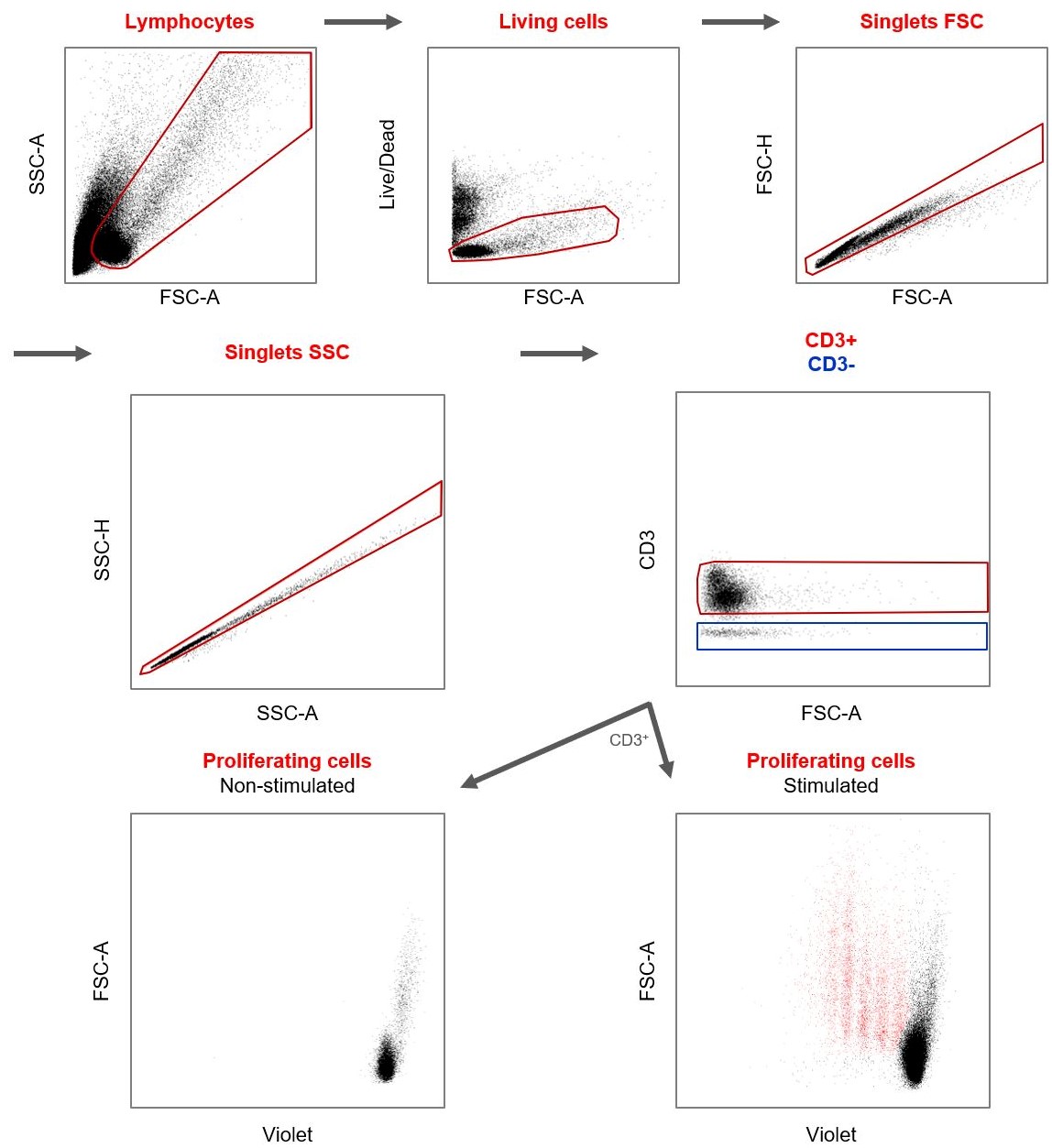

Supplement: Supplementary file 3 [file Image_3.jpeg]

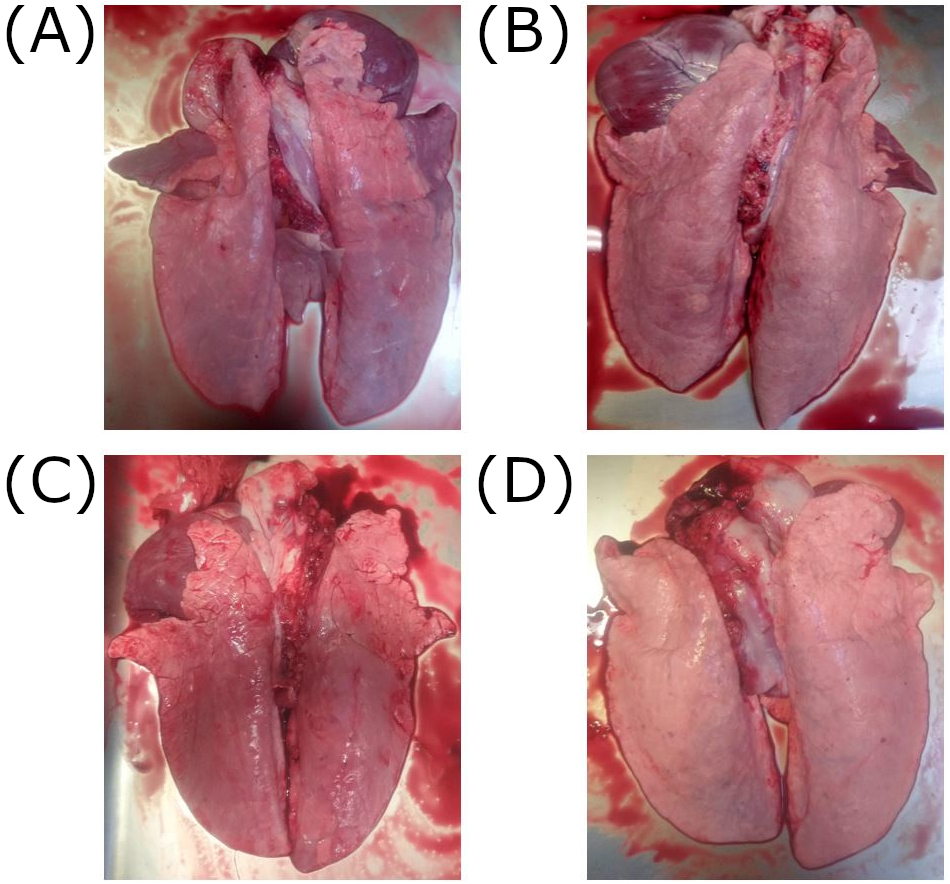

Supplement: Supplementary file 4 [file Image_4.jpeg]
