# Supplementary material for: Different local, innate and adaptive immune responses are induced by two commercial Mycoplasma hyopneumoniae bacterins and an adjuvant alone
Source: Front Immunol. 2022 Dec 7;13:1015525. doi: 10.3389/fimmu.2022.1015525 (PMC9768447; doi:10.3389/fimmu.2022.1015525)
Supplement: Supplementary file 5 [file DataSheet_1.docx]

Supplementary Material

Figure legends:

**Supplementary Figure 1: Threshold at fluorescence 2,500 to determine partition classification in the digital PCR assay.**

BAL samples were analyzed using a digital PCR assay targeting the P102 gene of *Mycoplasma hyopneumoniae*. After thermal cycling, fluorescence readout and a fluorescence spillover compensation matrix, small discrepancies in baseline fluorescence were corrected and a hard threshold was set at fluorescence 2,500 to allow partition classification.

**Supplementary Figure 2: Gating strategy to assess cytokine production by T cell subsets with CytExpert software.**

All plots shown are obtained from *M. hyopneumoniae*-stimulated T cells, isolated from piglets of V1 on D7. The cytokine production of CD8^+^, CD4^+^, CD4^+^CD8^+^ and CD4^-^CD8^-^ T cells is shown in the lower half of the figure. The rows represent the TNF-α, IFN-γ, TNF-αIFN-γ and IL-17A producing cells, respectively, within each T cell subset. For the TNF-αIFN-γ producing cells within each T cell subset, plots were pre-gated on TNF-α producing cells, as shown in the third row.

**Supplementary Figure 3: Gating strategy to assess proliferation by CD3^+^ T cells with CytExpert software.**

All plots shown are obtained from *M. hyopneumoniae*-stimulated T cells.

**Supplementary Figure 4: Lungs from animals with and without *Mycoplasma*-like lesions.**

The lungs of each animal were removed from the carcass at necropsy (D49) and scored for macroscopic *Mycoplasma*-like lung lesions. The score ranged between 0 (no *Mycoplasma*-like lesions) to 35 (entire lung affected). Representative pictures of lungs with (**A**, **B**) and without (**C**, **D**) *Mycoplasma*-like lesions are shown.

**Supplementary Table 1: LPS-induced IFN-γ, IL-1β and IL-6 secretion by blood monocytes.**

Piglets were injected on D0 with V1, A, V2 or physiological saline solution (control; C). Animals were challenge infected on D21 and euthanized on D49. The concentration of secreted IFN-γ, IL-1β and IL-6 (pg/mL) of LPS-stimulated blood monocytes from V1, A and C was analyzed with a Kruskal–Wallis H-test with Dunn-Bonferroni post hoc test. Results were considered significant when *p* ≤ 0.05. All results are expressed as the median (interquartile range).

| **Cytokine** | **Day** | **V1** | **A** | **C** | ***P* value** |
| --- | --- | --- | --- | --- | --- |
| IFN-γ  (pg/mL) | D0 | 1.25  (48.08) | 15.08  (54.41) | 4.37  (33.88) | 0.88 |
|  | D7 | 1.25  (0.00) | 1.25  (0.00) | 1.25  (0.00) | 0.33 |
|  | D21 | 25.00  (57.18) | 25.88  (62.55) | 4.42  (27.97) | 0.38 |
| IL-1β  (pg/mL) | D0 | 5227  (4634) | 4552  (4506) | 3430  (3707) | 0.10 |
|  | D7 | 4792  (5789) | 5277  (3489) | 8173  (10676) | 0.72 |
|  | D21 | 5266  (3962) | 6049  (4201) | 4997  (9119) | 0.93 |
| IL-6  (pg/mL) | D0 | 449  (373) | 342  (242) | 344  (135) | 0.89 |
|  | D7 | 827  (267) | 648  (460) | 774  (359) | 0.28 |
|  | D21 | 533  (415) | 491  (234) | 692  (419) | 0.18 |

**Supplementary Table 2: Vaccine efficacy parameters.**

Piglets were injected on D0 with V1, A, V2 or physiological saline solution (control; C). Animals were challenge infected on D21 and euthanized on D49. The sentinel group (S) was not included in the statistical analyses. For the percentage of lung area occupied by air (% Air), significant differences were analysed using ANOVA with a Tukey’s post hoc test. For the macroscopic lung lesion score (macro LL), a Kruskal–Wallis H-test with Dunn-Bonferroni post hoc test was used. The median microscopic lung lesion score (micro LL) was analyzed using an ordered logistic regression and an attempt was made to assess the influence of the vaccines and adjuvant on the respiratory disease score (RDS) using a generalized estimating equations procedure with an ordinal logit link function. Quasicomplete separation in the data prevented model convergence and further analysis was not possible for RDS. Statistical results were considered significant when *p* ≤ 0.05. For % Air, results are expressed as the mean ± standard deviation, while values for RDS, Macro LL and Micro LL are expressed as the median (interquartile range), except for the number of animals with at least one lobe with macro LL or with median micro LL > 2. NA = not analyzed

| **Parameter** | **Day/Period** | **S** | **V1** | **A** | **V2** | **C** | ***P* value** |
| --- | --- | --- | --- | --- | --- | --- | --- |
| RDS  (0 – 6) | D0 – D20 | 0  (0) | 0  (0) | 0  (0) | 0  (0) | 0  (0) | NA |
|  | D21 – D49 | 0  (0) | 0  (0) | 0  (1) | 0  (2) | 0  (2) | NA |
| Macro LL  (0 – 35) | D49 | 0.00  (0.13) | 0.00^a^  (0.00) | 2.31^b^  (7.79) | 2.14^b^  (5.34) | 6.29^b^  (7.04) | < 0.03 |
| # animals with at least one lobe with macro LL | D49 | 1/5 | 2/11 | 9/12 | 9/11 | 10/10 | NA |
| Micro LL  (0 – 5) | D49 | 2.03  (0.38) | 2.17^a^  (0.23) | 2.47^ab^  (0.64) | 2.23^a^  (0.47) | 2.97^b^  (0.28) | < 0.03 |
| # animals with median micro LL > 2 | D49 | 0/5 | 0/11 | 3/12 | 2/11 | 6/10 | NA |
| % Air | D49 | 33.23  ± 9.04 | 34.57  ± 7.39 | 32.29  ± 7.29 | 34.92  ± 7.49 | 31.03  ± 5.66 | 0.54 |
